# Amplified OTDR Systems for Multipoint Corrosion Monitoring

**DOI:** 10.3390/s120303438

**Published:** 2012-03-12

**Authors:** Jehan F. Nascimento, Marcionilo J. Silva, Isnaldo J. S. Coêlho, Eliel Cipriano, Joaquim F. Martins-Filho

**Affiliations:** 1 Polytechnic School of Pernambuco, University of Pernambuco (UPE), 52720-001, Recife, PE, Brazil; E-Mails: jehanfonseca@hotmail.com; 2 Department of Electronics and Systems, Federal University of Pernambuco (UFPE), 50740-550, Recife, PE, Brazil; 3 Department of Electrical Engineering, Federal University of São Francisco Valley (UNIVASF), 56305-971, Petrolina, PE, Brazil; E-Mail: isnaldo.coelho@univasf.edu.br

**Keywords:** corrosion, optical fiber, OTDR, fiber sensor, optical amplifier, EDFA

## Abstract

We present two configurations of an amplified fiber-optic-based corrosion sensor using the optical time domain reflectometry (OTDR) technique as the interrogation method. The sensor system is multipoint, self-referenced, has no moving parts and can measure the corrosion rate several kilometers away from the OTDR equipment. The first OTDR monitoring system employs a remotely pumped in-line EDFA and it is used to evaluate the increase in system reach compared to a non-amplified configuration. The other amplified monitoring system uses an EDFA in booster configuration and we perform corrosion measurements and evaluations of system sensitivity to amplifier gain variations. Our experimental results obtained under controlled laboratory conditions show the advantages of the amplified system in terms of longer system reach with better spatial resolution, and also that the corrosion measurements obtained from our system are not sensitive to 3 dB gain variations.

## 1. Introduction

Sensors based on optical fibers are used to monitor various measurands such as temperature, pressure, corrosion, humidity, pollution, current, voltage, electric field, magnetic field and others [1–3]. The optical fiber-based monitoring techniques have certain advantages, such as simplicity, versatility, safety, low weight and reliability. They are also considered immune to external electromagnetic noise. Optical fibers can carry light signals over long distances without appreciable loss of propagation. The use of sensors based on optical fibers has a special value in hostile environments such as inside electrical machinery, in areas exposed to high electromagnetic fields (e.g., within high power transformers) and in places of difficult access and subject to high temperature and pressure, as in pipelines and oil wells [4,5].

Corrosion monitoring is an important aspect of modern infrastructure in industry sectors such as mining, aircraft, shipping, oilfields, as well as in military and civil facilities [4]. Optical fiber-based corrosion sensors have been investigated in recent years mainly because of the advantages achieved by the use of optical fibers, as already pointed out. Recent reviews of the technologies employed in fiber-based corrosion sensors can be found in López-Higuera *et al.* [3], Wade *et al.* [6], Wang and Huang [7], and in Martins-Filho and Fontana [5]. The reported applications include corrosion monitoring in aircraft [8], in the concrete of roadways and bridges [9] and in oilfields [5] among others.

Martins-Filho *et al.* have presented for the first time the concept and the experimental results of a fibre-optic-based corrosion sensor using the optical time domain reflectometry (OTDR) technique as the interrogation method [5,10]. Our proposed sensor system consists of several sensor heads connected to commercial OTDR equipment by single-mode optical fibers and fiber couplers. The couplers split the optical signal such that a small fraction is directed to the sensing heads. The sensor head consists of a cleaved-end fiber where a metal film is deposited. As the metal film is removed from the fiber facet due to corrosion, the reflected light measured in the OTDR decreases. The proposed sensor system is multipoint, self-referenced, has no moving parts and can detect the corrosion rates several kilometers away from the OTDR equipment [5]. However, the system reach is limited by the OTDR dynamic range due to the insertion losses of the optical couplers and fiber losses.

Optical amplifiers are employed in optical communication systems to obtain high-capacity and long distance data transmission [11]. These sub-systems are capable of regenerating the optical signal, which has been attenuated along the fiber. Among the several different optical amplification technologies, the Erbium Doped Fiber Amplifier (EDFA) is the most mature, low cost and widely used method for different applications. EDFAs can provide amplification in the spectral band from 1,530 nm to 1,560 nm [11] and there are many reports of their use along with an OTDR for sensor applications [12–14].

In this paper we present experimental results for two configurations of an amplified OTDR corrosion sensor system that employs an EDFA to achieve longer system reach. Our results show the advantages of the amplified system in terms of longer system reach with better spatial resolution. To our knowledge, this is the first report on the experimental characterization of amplified OTDR systems for corrosion sensor applications, and also the first to evaluate the sensitivity of the measurements to amplifier gain variations. In Section 2 we present the OTDR monitoring system with a remotely pumped in-line EDFA to evaluate the increase in system reach compared to a non-amplified configuration. In Section 3 we present the monitoring system with an EDFA in booster configuration to perform corrosion measurements and evaluate the system sensitivity to amplifier gain variations. In Section 4 we present our conclusions.

## 2. Increasing the Monitoring Distance

**Figure 1.**
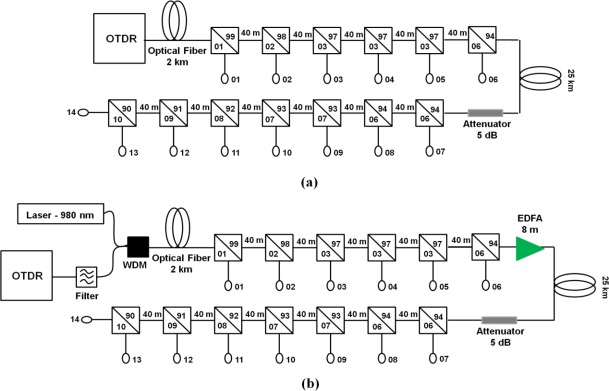
Multipoint sensor system (**a**) without and (**b**) with amplification (remotely pumped in-line EDFA).

Figure 1(a) shows the multipoint sensor system with 14 sensor heads, numbered from 1 to 14. It consists of an Optical Time Domain Reflectometer (OTDR Anritsu 9076B) operating at a wavelength of 1.55 μm, connected to a single mode fiber (2 km long), some optical couplers, an additional single mode fiber (25 km long, approximately) and a 5 dB attenuator. The couplers split the optical signal emitted by the OTDR so that a small fraction (1–10%) is directed onto the sensor heads. For the sake of simplicity, in this section the sensor heads are bare end cleaved fibers. The signals reflected by the sensor heads are detected by the OTDR as peaks in the OTDR trace. The 25 km long fiber and the optical attenuator were placed between the 6th and the 7th sensor head to simulate a situation where we have a first set of sensors placed closer to the OTDR and another set of sensors placed much further away. This is a typical situation where the long distance and the excess loss from the couplers can limit the system reach, making it difficult to obtain results for the sensor heads at the end of the system.

Figure 1(b) presents the amplified version of the sensor system shown in Figure 1(a). An 8 m long Erbium Doped Fiber (EDF) is placed after the 6th head to amplify the OTDR laser signal. The EDF (3M part number FS-ER-7A28) is remotely pumped by a 980 nm multimode laser that is inserted in the system close to the OTDR by a proper WDM coupler. We found that the insertion loss of the couplers in the pump laser wavelength is small, whereas the attenuation is 0.78 dB/km in the standard fiber, such that for 100 mW pump power at laser output, about 17 mW pump power is launched in the EDF. Only the signals from the sensor heads after the EDF (heads 7 to 14) are amplified. Figure 1(b) also shows an optical band-pass filter close to the OTDR. It is centered at the OTDR emitting wavelength, around 1.55 μm, and it is employed to reduce the amplified spontaneous emission (ASE) power that comes from the EDF entering into the OTDR, which can cause its saturation [15,16]. The filter bandwidth is 1 nm FWHM, whereas the OTDR laser linewidth is 10 nm. This difference means that the filter attenuates the OTDR signal. The obtained net gain (EDFA gain minus filter attenuation) is 10 dB.

**Figure 2.**
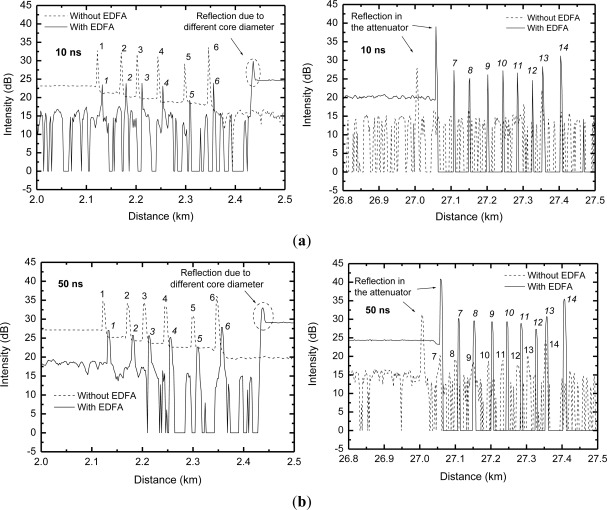
OTDR traces of the amplified (solid line) and non-amplified (dashed line) multipoint sensor system for OTDR pulsewidth of (**a**) 10 ns and (**b**) 50 ns. Pump power at EDF input is 17 mW.

In Figure 2 we present the OTDR traces obtained from the experimental apparatus shown in Figure 1(a) (dashed line) and Figure 1(b) (solid line) for 10 ns and 50 ns OTDR pulsewidths. In the left hand side graphs one can see six peaks corresponding to the first six sensor heads of the systems, whereas the right hand side graphs show the remaining eight sensor heads, numbered as in Figure 1. Additional peaks can be seen, which are due to reflections in the interface between different fibers or due to the optical attenuator inserted in the system. Note that the first six sensor heads are not amplified, therefore they only experience attenuation due to the bandpass filter in the amplified system (Figure 1(b)). This explains why they present lower intensity OTDR traces in the amplified scheme than in the non-amplified scheme. On the other hand, the opposite behavior can be observed in the last eight sensor heads, *i.e*., the amplified scheme presents higher intensity OTDR traces, since the sensor heads experience the EDFA gain.

Figure 2(a) clearly show that for 10 ns pulsewidth the OTDR sensor system cannot distinguish the peaks of the heads number 7 to 14 from the background noise. These sensor heads are beyond the system reach, which depends on the OTDR equipment dynamic range. However, in the amplified system, for the same experimental conditions, one can clearly see the peaks of the last eight sensor heads of the system. This is an indication that the use of an EDFA can extend the reach of the OTDR system. The actual increase in system reach depends on the insertion loss of each element in the system (couplers and optical fiber).

Figure 2(b) show a similar experiment, but with the OTDR equipment set to produce 50 ns pulses, instead of 10 ns pulses as in the case of Figure 2(a). Wider optical pulses imply more photons per pulse, which means that the OTDR has a higher dynamic range and the system has a longer reach. However, wider pulses also imply lower spatial resolution of the OTDR traces [17]. Indeed, differently from the case of 10 ns pulses, in this situation the peaks numbered 7 to 14 can be observed just above the background noise, even in the non-amplified experiment. The amplified OTDR trace shows these peaks 10 dB higher. Previous studies showed that the EDFA presented no variation of gain for OTDR pulsewidths between 10 ns and 100 ns [18].

## 3. Corrosion Measurements with the Amplified System

**Figure 3.**
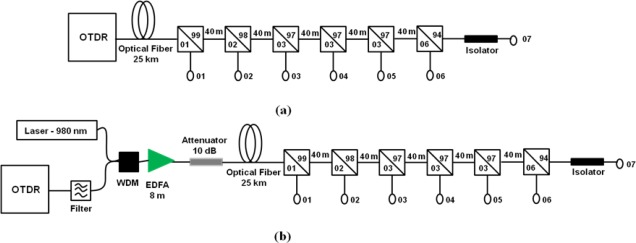
Multipoint corrosion sensor system (**a**) without and (**b**) with amplification (booster EDFA).

In order to verify the effects of the amplification scheme and the EDFA gain variations on the corrosion monitoring results we setup the configurations shown in Figure 3. It shows a multipoint corrosion sensor system without (Figure 3(a)) and with (Figure 3(b)), amplification. Differently from the configuration shown in Figure 1, where the amplifier is placed in line, among the sensor heads, in Figure 3 the amplifier is placed close to the OTDR, as booster to its signal, before the sensor heads. In the setup of Figure 3 we placed the 25 km long fiber reel between the sensor heads and the OTDR with EDFA arrangement. We choose arbitrarily the 5th sensor head to perform corrosion measurements. This sensor head has 39 nm of aluminum deposited on a cleaved fiber facet by a standard thermal evaporation process. We put an isolator at the end of the system (7th sensor head) to avoid its reflection back into the OTDR, which could cause OTDR saturation. We also had to use a 10 dB attenuator in the amplified system shown in Figure 3(b). This was to avoid the saturation of the OTDR, which is due to the high intensities reflected from the first sensor heads. This effect shows a clear disadvantage of this booster amplification scheme compared to the in line scheme shown in Figure 1(b). In the inline scheme the amplification takes place where the signal has low power, after several sensor heads, whereas in the booster scheme the amplification occurs close to the OTDR emission, where the signal has high power. The excess signal power reflected from the first sensor heads cause the OTDR saturation. Since a 10 dB attenuator corresponds to 50 km of standard single mode fiber (0.2 dB/km), the booster amplification scheme would be useful for ultra-long distances between OTDR and sensor heads.

**Figure 4.**
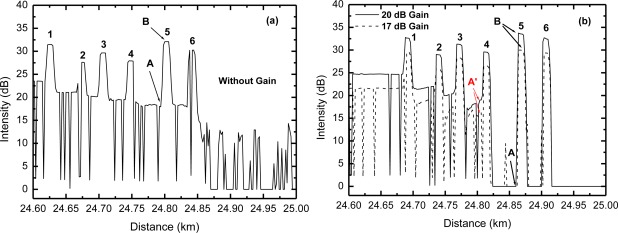
OTDR traces for the non-amplified (**a**) and amplified (**b**) multipoint sensor system of Figure 3 for different gains (17 dB and 20 dB).

In Figure 4 we present the OTDR traces obtained from the experimental apparatus shown in Figure 3(a,b), where the numbered peaks correspond to sensor heads numbered from 1 to 6 in Figure 3. Moreover, Figure 3(b) shows results for two different EDFA gain values, 17 dB (dashed line) and 20 dB (solid line), obtained by changing the laser pump power at EDF, from 17 mW to 25 mW, respectively. For these measurements we used the OTDR pulsewidth of 50 ns. In a previous work [18] we have shown that the OTDR pulsewidth has no effect on the measurements of system gain. We also obtained the gain *versus* pump power curve and we found that for 25 mW pump power the EDFA reached its maximum gain of 20 dB [18].

Note from Figure 4(b) that after the 4th sensor head the OTDR traces go to zero between the peaks of the sensor heads. This behavior is not observed in Figure 4(a), for the non-amplified scheme, where the Rayleigh backscattering level can be observed until the 6th sensor head (end of the system). We believe this may be due to the ASE noise power generated by the EDFA, that screens the Rayleigh backscattered power generated at the fiber further away from the OTDR.

**Figure 5.**
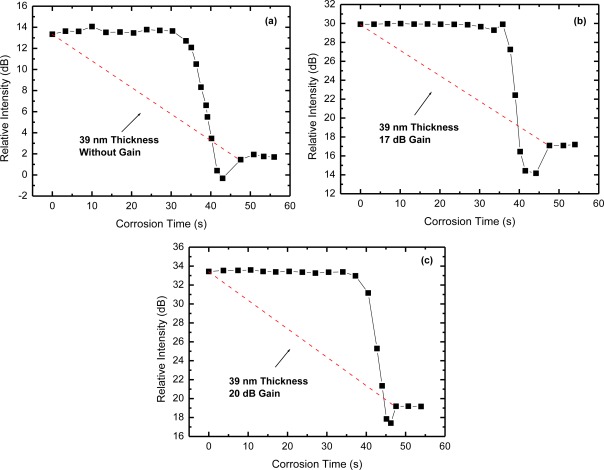
OTDR relative intensity as a function of the Al film corrosion time for the following cases: (**a**) without gain; (**b**) 17 dB gain; and (**c**) 20 dB gain.

In Figure 5 we show the temporal evolution of the OTDR trace as the corrosion process takes place on the sensor head number 5, which has 39 nm thick aluminum film deposited on the fiber facet by standard thermal evaporation. We used the Al-etcher 25 H_3_PO_4_: 1 HNO_3_: 5 CH_3_COOH, which is used in micro device fabrication processes and it has a corrosion rate of Al of 50 nm/min [19]. We used aluminum as the metal for the sensor head and this Al-etcher because of its known etch rate, which is suitable for laboratory experiments. For real applications different metals or other materials should be used in the sensor head to better match to the monitored structure. The sensor head number 5 is dipped in the Al-etcher for short time intervals and the OTDR trace is obtained after each one. As the aluminum is being removed from the fiber facet the reflected light measured in the OTDR decreases. Figure 5 shows the ratio of peak (point B) to valley (point A) of the reflected light shown in Figure 4 as a function of the aluminum corrosion time. Figure 5 shows that up to 30 s of corrosion there is no significant change in the OTDR measured reflected light, since the aluminum is still too thick. Further up from this point the reflection drops to a minimum and then stabilizes at a constant level. The constant level means that the corrosion process on the fiber facet has ended. We obtain the corrosion rate by taking the deposited metal thickness and the time taken to reach the constant level. In Figure 5(a) we show the results for the non-amplified scheme of Figure 3(a), whereas in Figure 5(b,c) we show the results for the amplified scheme shown in Figure 3(b) with 17 dB and 20 dB gain, respectively. In all of them the corrosion of the 39 nm Al film took 47 s, which corresponds to the expected corrosion rate of 50 nm/min. The valley shown in Figure 5(a–c), just before the end of the corrosion process, is a feature that has been preliminarily discussed [5] and will be the subject of future work.

**Figure 6.**
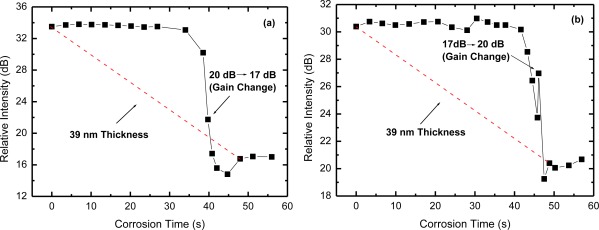
OTDR relative intensity (B-A) for the corrosion of the Al film under gain changes: (**a**) from 20 dB to 17 dB and (**b**) from 17 dB to 20 dB.

**Figure 7.**
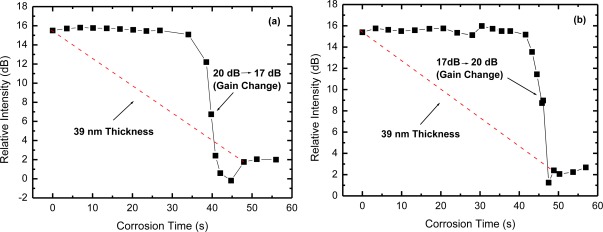
OTDR relative intensity (B-A’) for the corrosion of the Al film under gain changes: (**a**) from 20 dB to 17 dB and (**b**) from 17 dB to 20 dB.

We also investigated the effect of EDFA gain variations that may occur between two consecutive measurements of the OTDR trace evolution. These variations may occur in real applications due to changes in the laser pump power (aging effect), or even due to changes in EDF temperature [11]. Figure 6(a) shows measurements of the OTDR relative intensity as a function of corrosion time with a change in EDFA gain, from 20 dB to 17 dB, in the indicated point. In Figure 6(b) the gain changes from 17 dB to 20 dB. As could be expected, the measured corrosion time was not altered by the changes in EDFA gain. However, we can observe, especially in Figure 6(b), that there was a peak in the curve due to the gain change. Note that Figure 6 is obtained as the ratio of point B and point A (B-A) shown in Figure 4(b). The point B is the reflection level from the sensor head and it is sensitive to gain changes, but point A, which is a reference level, is not, because it is zero. Therefore, this sensitivity to gain changes could be minimized or even removed if another reference level is used, a reference that is also affected by the gain change. For example, the point A’ shown in Figure 4(b) is also affected by the gain changes, similarly to the point B. If we recalculate the OTDR relative intensities of Figure 6 using the point A’ instead of point A, we obtain the curves shown in Figure 7(a,b), which show almost no sign of gain changes. Therefore, the proper choice of the reference level to obtain the relative intensities is important to guarantee the immunity of the obtained results to signal level changes, *i.e*., to keep it self referenced.

## 4. Conclusions

We have presented experimental results for two configurations of an amplified OTDR system for multipoint corrosion sensors, one with the remotely pumped EDFA placed among the sensor heads, in the fiber line, away from the OTDR equipment, and the other with the EDFA placed close to the OTDR equipment, in a booster configuration, before the sensor heads. Our results indicated that the use of the booster configuration is more indicated for the case of very long distance measurements, several tens of kilometers distance from the OTDR equipment and the first sensor head. We also showed that the optical amplification increased the system reach, allowing for the increase of the number of sensor heads in the system and the use shorter OTDR pulsewidth, which implies in better spatial resolution for the monitoring system. We also performed corrosion measurements with the amplified and non-amplified systems, under controlled laboratory conditions, for different amplifier gain values. Our results showed that the corrosion measurements obtained from our system are not sensitive to 3 dB gain variations. Moreover, if a suitable reference level is chosen, the gain variations that may occur during the course of the measurements can be mitigated, which reinforces the self-referenced characteristic of our fiber-optic sensor system. To our knowledge, this is the first report on the experimental characterization of amplified OTDR systems for long distance corrosion sensor applications.

We believe the amplified OTDR system may be improved by the use of an EDFA pump laser operating at 1,470 nm, instead of the 980 nm, because the longer wavelength has lower fiber attenuation, which would improve on the power efficiency of the inline remotely pumped EDFA.

Our sensor system is multipoint, self-referenced, has no moving parts (all-fibre) and can detect corrosion rates for each head several kilometres away from the OTDR, thus making the system ideal for “in-the-field” continuous monitoring of corrosion and erosion. This system may have applications in harsh environments and long distances, such as in deepwater oil wells and gas flowlines (including from the presalt region). Different materials can be deposited on the fibre facet to better match to the materials under corrosion/erosion. This system may enable inferred condition-based maintenance without production interruption, decreasing the cost of oil production, and substantially reducing the risk of environmental disasters due to the failure of unmonitored flowlines.
